# Anomalous *in situ* Activation of Carbon-Supported Ni_2_P Nanoparticles for Oxygen Evolving Electrocatalysis in Alkaline Media

**DOI:** 10.1038/s41598-017-08296-0

**Published:** 2017-08-15

**Authors:** Young-Hoon Chung, Injoon Jang, Jue-Hyuk Jang, Hyun S. Park, Hyung Chul Ham, Jong Hyun Jang, Yong-Kul Lee, Sung Jong Yoo

**Affiliations:** 1 Fuel Cell Research Center, Korea Institute of Science and Technology (KIST), 02792 Seoul, Republic of Korea; 2 Applied Materials Examination Division, Korean Intellectual Property Office (KIPO), 35208 Daejeon, Republic of Korea; 3 Green School, Korea University, 02841 Seoul, Republic of Korea; 4 Department of Chemical Engineering, Dankook University, 16890 Yongin, Republic of Korea; 5 School of Chemical and Biological Engineering, Seoul National University (SNU), 08826 Seoul, Republic of Korea; 6 Division of Energy & Environment Technology, KIST-School, Korea University of Science and Technology, Daejeon, 34113 Republic of Korea

## Abstract

Electrochemical water splitting is one of the most promising systems by which to store energy produced from sustainable sources, such as solar and wind energy. Designing robust and stable electrocatalysts is urgently needed because of the relatively sluggish kinetics of the anodic reaction, *i*.*e*. the oxygen evolution reaction (OER). In this study, we investigate the anomalous *in situ* activation behaviour of carbon-supported Ni_2_P nanoparticles (Ni_2_P/C) during OER catalysis in alkaline media. The activated Ni_2_P/C shows an exceptionally high activity and stability under OER conditions in which the overpotential needed to achieve 10 mA cm^−2^ was reduced from approximately 350 mV to approximately 300 mV after 8,000 cyclic voltammetric scans. *In situ* and *ex situ* characterizations indicate that the activity enhancement of Ni_2_P catalysts is due to a favourable phase transformation of the Ni centre to *β*-NiOOH, including increases in the active area induced by structural deformation under the OER conditions. These findings provide new insights towards designing transition metal/phosphide-based materials for an efficient water splitting catalyst.

## Introduction

The electrochemical splitting of water (H_2_O → H_2_ + 1/2O_2_) has been extensively studied given its promise towards achieving renewable solar-to-chemical fuel conversion^1, 2^. This process consists of two reactions: the hydrogen evolution reaction (HER) and the oxygen evolution reaction (OER), both of which require electrocatalysts that significantly reduce the overpotentials. This is particularly true for the OER, given its much more sluggish kinetics^3, 4^. Unfortunately, many OER catalysts that show the necessary performance are based on expensive precious metals, such as Ru and Ir^3, 5^. Other catalysts have been tested to reduce costs; these catalysts are based on more abundant metals but are nonetheless active and durable, and include first-row transition metal complexes centred on Ni^6–15^, Co^16–19^, Mn^18^, Ni-Fe^20, 21^, and Ni-Co-Fe^22^.

**Figure 1:**
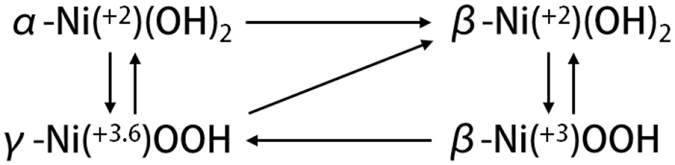
Phase transformation between different Ni(OH)_2_ and NiOOH species.

Ni-based electrocatalysts are among the most extensively studied materials given their high activity in alkaline media^4^. Under OER conditions, an active Ni centre changes oxidation states between Ni(OH)_2_ and NiOOH during the potential scans. According to the Bode diagram^23, 24^, the phase transformation among different Ni(OH)_2_ and NiOOH species could proceed in alkaline media (Fig. 1). One of the most important aspects of designing a robust catalyst is enhancing the intrinsic activity^7, 8, 12, 13, 20–22^. While still uncertain, the *β*-NiOOH phase is generally considered the most active towards the OER among the investigated Ni species^19, 20, 25^. The Bell group posited that the reaction rate is proportional to the *β*-NiOOH content and demonstrated a reduced overpotential in Ni-Fe oxide catalysts^21^. Meanwhile, Li and Selloni predicted that *β*-NiOOH-based structures would demonstrate smaller OER overpotentials than their *γ*-NiOOH equivalents^26^. The overall catalysis efficiency suggests that maximizing the active surface area is also an important parameter^6, 7, 10^. Recently, Nardi *et al*. exhibited that the OER electrocatalysis of a NiO film produced through atomic layer deposition can be improved by increasing the number of accessible Ni sites^7^. Likewise, Stern *et al*. found an enhanced catalytic activity for nano-sized NiO_*x*_ and Ni(OH)_2_ with a higher number of active sites^10^.

A few studies concerning transition metal-P compounds as OER catalysts have been conducted. Kanan and Nocera reported *in situ* formation of a Co-Pi oxygen-evolving complex (OEC) film that was electrochemically active in an aqueous phosphate buffer solution^19^. Recently, nickel phosphides were used as efficient oxygen evolving catalysts^11–15^. In these cases, the formation of a NiO_*x*_ layer on the surface, which contributes to the enhanced OER performance, might be associated with the underlying P. However, the mechanism of this enhancement is not yet fully understood.

Herein, we investigate the use of Ni_2_P nanoparticles on conductive carbon supports with a high surface area for OER catalysis. We observed anomalous *in situ* activation of the electrocatalyst, resulting in an exceptionally high activity and durability. We also revealed that these unique properties are related to the continuous phase transition of Ni species to *β*-NiOOH, including structural deformation. This finding provides a new method by which to produce a robust and durable Ni_2_P-based OER electrocatalyst starting from metal phosphide nanoparticles.

## Results

**Figure 2:**
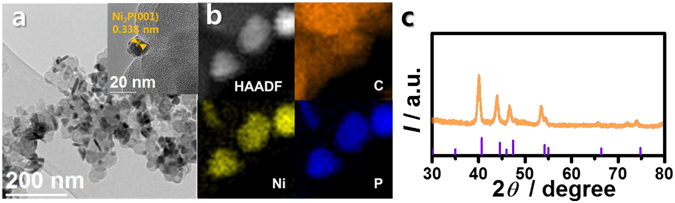
(**a**) Transmission electron microscopy (TEM) images of Ni_2_P/C, (**b**) high-angle annular dark field (HAADF, left) image and corresponding elemental mappings (C, Ni, and P) of Ni_2_P/C using energy dispersive X-ray spectroscopy (EDS), and (**c**) X-ray diffraction (XRD) pattern of Ni_2_P/C (purple line: JCPDS 03-0953).

The Ni_2_P nanoparticles were loaded onto the catalyst (Ni_2_P/C) to achieve a sufficient dispersity and conductivity. The total phosphide loading was set to 50 wt% (which was the highest possible value) to minimize carbon corrosion reactions under the OER conditions by decreasing the exposure of bare carbon surfaces. As shown in Fig. 2a, the Ni_2_P loading on the carbon supports was successful; the high-resolution transmission electron microscopy (HR-TEM) image clearly shows a lattice distance typical of the Ni_2_P (001) plane^13^. Meanwhile, energy dispersive X-ray spectroscopy (EDS) under scanning TEM (STEM) mode demonstrated a good dispersion in the bulk particles (Fig. 2b). The X-ray diffraction (XRD) pattern (Fig. 2c) also confirms the formation of a typical Ni_2_P structure, with peaks at 40.8°, 44.6°, 47.3°, 54.2°, and 54.4° corresponding to the (111), (021), (210), (300), and (002) planes, respectively (JCPDS 03-0953). Overall, the as-prepared particulate-shaped Ni_2_P/C and Ni_2_P were clearly present on the carbon surface.

**Figure 3:**
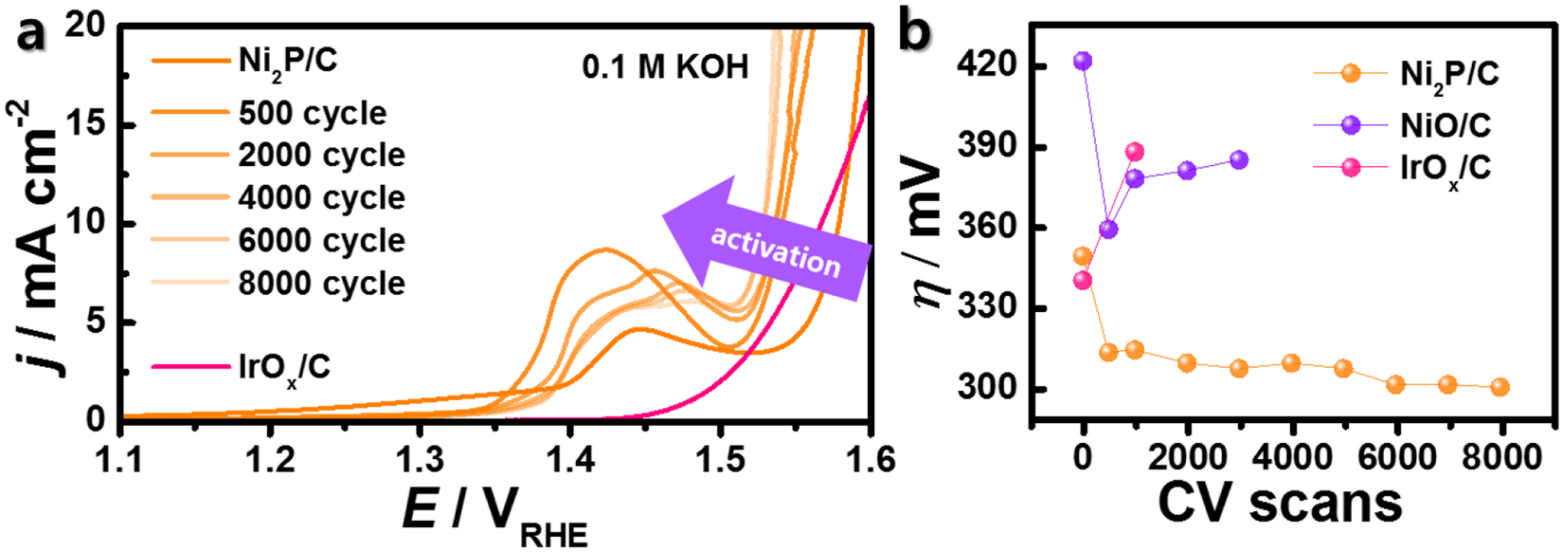
(**a**) Ohmic resistance-compensated polarization curves of the oxygen evolution reaction (OER) for Ni_2_P/C, and (**b**) overpotentials (*η*) at 10 mA cm^−2^ of Ni_2_P/C and NiO/C with respect to the potential cycles. All measurements were performed in 0.1 M KOH saturated by high purity oxygen (99.999%) at room temperature.

The OER electrocatalytic activities of Ni_2_P/C, NiO/C, and IrO_*x*_/C were investigated using a glassy carbon electrode (area: 0.196 cm^2^) in a 0.1 M KOH aqueous solution. Commercial carbon-supported NiO and IrO_*x*_ nanoparticles (hereafter denoted as NiO/C and IrO_*x*_/C, respectively) were used for comparisons to our materials. Interestingly, the oxygen production currents increased with consecutive cyclic voltammetric (CV) scans, as shown in the OER polarization curves (Fig. 3a). For example, the initial OER activity at *j* = 10 mA cm^−2^ was measured at about 350 mV, which is slightly more sluggish than the value of 340 mV for IrO_*x*_/C (one of the most active OER catalysts in basic aqueous solutions)^3, 5^. However, the enhanced OER activity was observed over time, even while the electrocatalytic activity of IrO_*x*_/C and NiO/C significantly slowed (Supplementary Figs S1 and S2). After 8,000 CV scans, an overpotential of about 300 mV was needed to achieve a current density of 10 mA cm^−2^ with the Ni_2_P/C electrocatalyst (*E* = 1.53 V_RHE_; RHE = reversible hydrogen electrode), while IrO_*x*_/C and NiO/C initially demanded values of 340 and 420 mV, respectively. According to Ni2p XPS spectra of Ni_2_P and Ni_2_P/C as shown in Supplementary Fig. S12, both spectra showed almost identical peak position. Therefore, supported carbon does not affect to the active materials and there is no synergistic effect between carbon and Ni_2_P. Further, the mass-normalized current density of the Ni_2_P/C electrocatalyst (64.9 A g^−1^ at 1.53 V_RHE_) was improved by factors of about 2 and 19 with respect to that of IrO_*x*_/C (30.5 A g^−1^) and NiO/C (3.4 A g^−1^), respectively (Supplementary Fig. S2). Note that the observed overpotentials for the activated Ni_2_P/C electrocatalysts are among the lowest values reported under these conditions (Supplementary Table S1)^6, 9, 14, 16–18, 20, 22, 27–29^.

Trace amounts of Fe incorporated into the Ni(OH)_2_ catalysts can enhance the OER electrocatalysis in the first few potential cycles through the formation of highly active Ni-Fe-O species, as previously reported^30^. Because the removal of a trace amount Fe in the electrolyte is almost impossible in practical applications, we now clarify that the prominent OER activity of Ni_2_P/C is derived from either incorporation of Fe in oxygenated Ni species or is an intrinsic property of Ni_2_P/C itself. In fact, NiO/C also showed an improved performance with overpotentials decreasing from about 420 to 360 mV after 500 cycles, as shown in Fig. 3 and Supplementary Fig. S2. However, this improvement was not sustained over additional cycles in the harsh electrochemical environment employed for testing; the NiO/C overpotential later increased to 390 mV after 3,000 scans, which is in sharp contrast to the continued decrease in the corresponding Ni_2_P/C value up to 8,000 trials. Once the trace Fe was removed, the enhanced activation behaviour for Ni_2_P/C was only detectable over an extended period of time, whereas the NiO/C performance decreased immediately (Supplementary Fig. S3 and Supplementary Fig. S4), which is consistent with the literature^7, 10, 21, 30^. Considering that the effect of Fe incorporation is eliminated by the experiments using purified solutions, the activation process would be explained only by changes in the inherent properties of Ni_2_P/C, such as *in situ* activation during the electrochemical cycles derived from continuous structural modifications, as discussed later.

## Discussion

**Figure 4:**
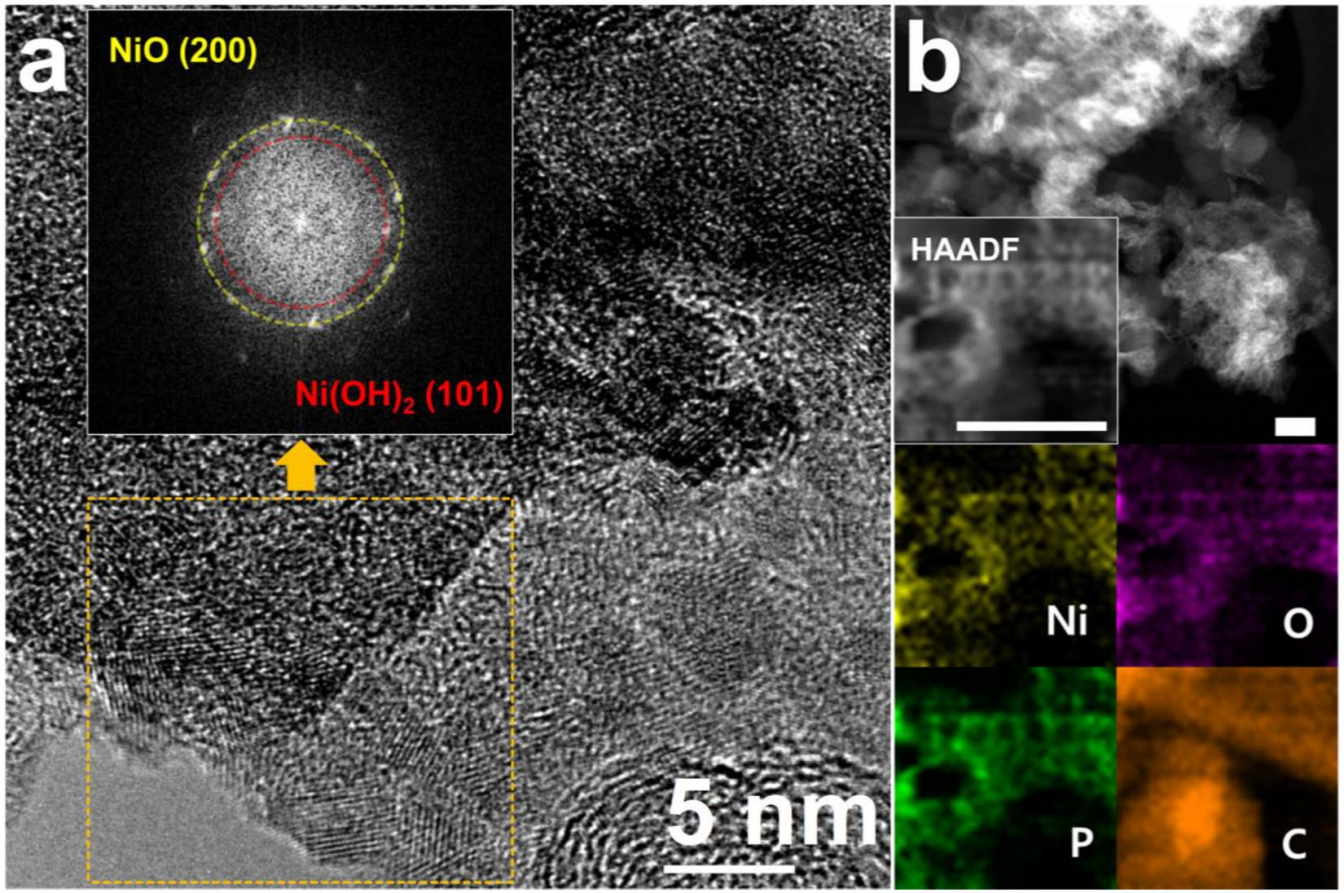
(**a**) High-resolution TEM image of Ni-O (orange dotted square) with a corresponding fast Fourier transform (FFT) pattern (inset figure), and (**b**) high-angle annular dark field (HAADF) images and elemental mapping using energy dispersive X-ray spectroscopy (EDS).

**Figure 5:**
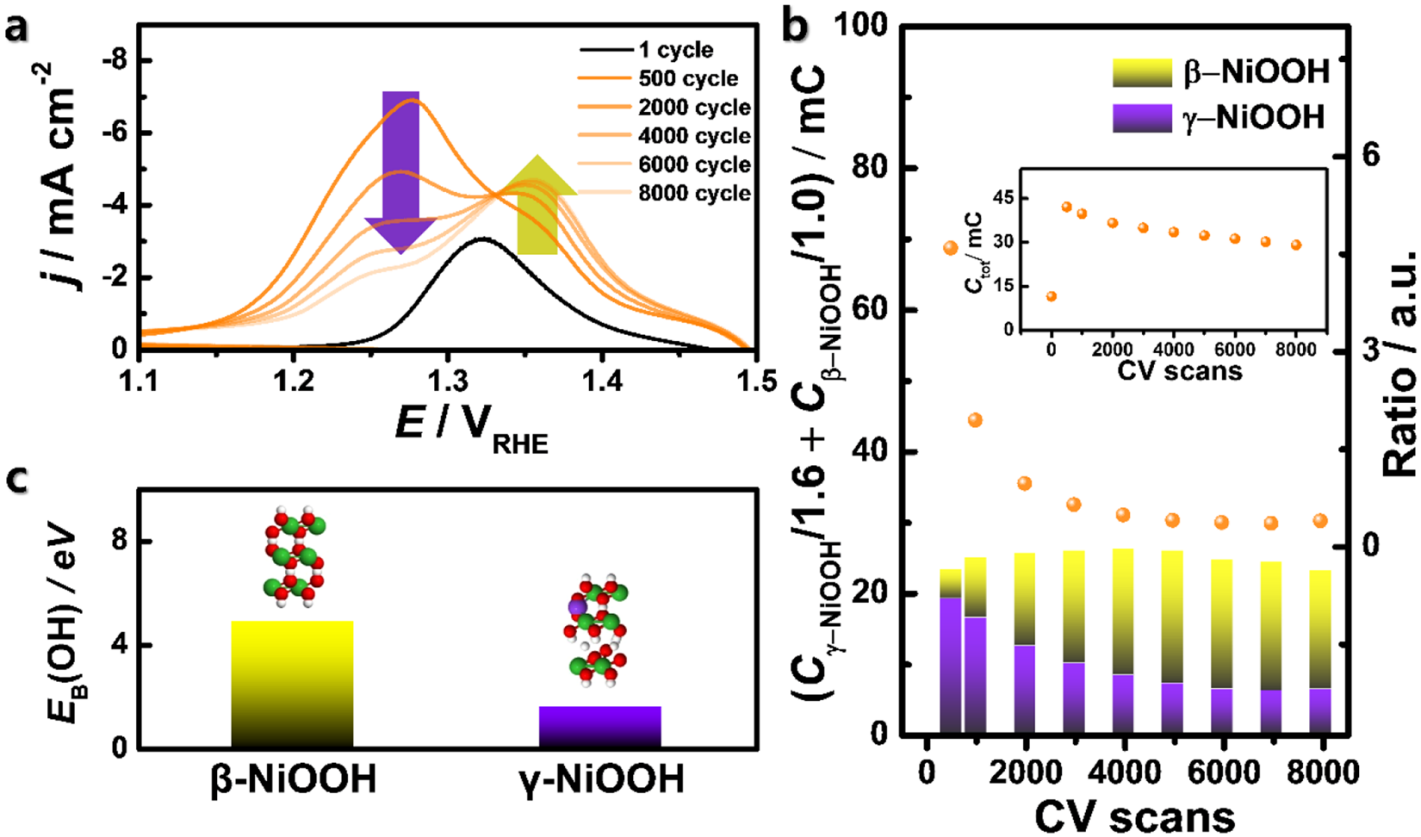
(**a**) Cyclic voltammograms (CVs) of Ni_2_P/C at the negative scans, (**b**) normalized charges assigned to the reduction current related to *β*-NiOOH and *γ*-NiOOH, estimated by the deconvolution of the CVs in (**a**) by considering different electron transfer numbers (shown in Fig. 1). Inset: calculated total reduction charges of the CVs in (**a**) and the estimated molar ratio of *γ*-NiOOH to *β*-NiOOH in repeated potential cycles, and (**c**) binding energy of OH (*E*_B_(OH)) for *β*-NiOOH and *γ*-NiOOH, calculated by density functional theory.

We analysed the catalyst after the electrochemical measurements to clarify the source of the anomalous activation behaviour. As shown in the TEM images in Fig. 4a, the Ni_2_P nanoparticles were deformed during the OER, coating a film-like structure onto the carbon support. This is a marked difference from the original composite displayed in Fig. 2a. The high-resolution images show very thin, small crystalline structures, as indicated by the FFT patterns of NiO and Ni(OH)_2_. Meanwhile, the high-angle annular dark field (HAADF) images using STEM show that relatively heavy atoms are still placed on the carbon supports (Fig. 3b). Likewise, the EDS data indicate that Ni, P, and O are well dispersed on the carbon surface. Thus, the very thin, newly evolved Ni-O-P layer on the carbon supports resulted from electrocatalysis. In Ni_2_P/C, because the carbonaceous materials with high surface were used as supports, the exact active surface area cannot be measured by conventional physicochemical methods such as Brunauer–Emmett–Teller (BET) measurements. Alternatively, we used the area of reduction peak of Ni(II)/Ni(III), directly associated with the active surface of nickel-based materials. The corresponding increase in the surface area likely accounts for the improved electrochemical performance, a conclusion that was confirmed by observing the rapid growth in electrochemical performance after 500 CV scans, as estimated by the area of characteristic Ni(II)/Ni(III) peaks (Fig. 5a and b)^7^. In addition, the outstanding OER activity might be attributed to the improved conductivity and charge transfer capability because of the incorporation of carbon supports into nickel phosphides during the structural deformation^14^.

**Figure 6:**
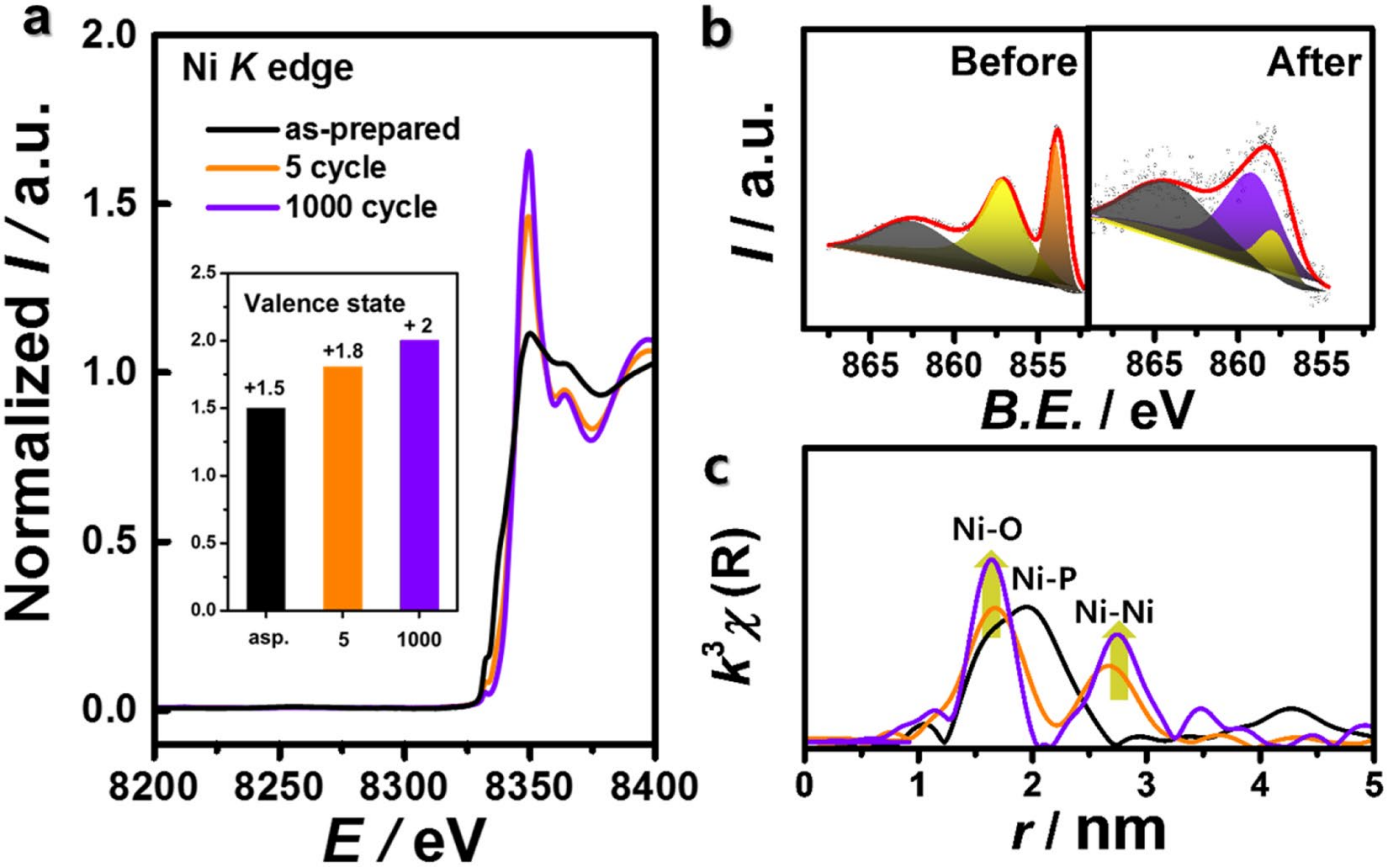
(**a**) X-ray absorption near-edge spectra (XANES) of the Ni *K* edge (*E*_0_ = 8333 eV) with a valence states (inset figure), (**b**) X-ray photoelectron spectra (XPS) of the Ni 2*p* level before (left) and after the OER (right), and (**c**) extended X-ray absorption fine structure (EXAFS) and structural correlation of Ni_2_P/C after the OER.

Next, X-ray absorption and emission spectroscopy was used to gather detailed structural information (Fig. 6). The initial Ni_2_P/C structure was completely deformed after five CV scans. X-ray absorption near-edge spectroscopy (XANES) showed that the Ni *K* edge shifted largely in the positive direction during the first five scans, with additional changes through 1,000 scans (Fig. 6a). This demonstrates progressive oxidation, especially when combined with the X-ray photoelectron spectroscopy (XPS) measurements of the Ni 2*p* core-level (Fig. 6b). The initially prepared Ni_2_P/C showed a typical Ni-P peak (orange) and a surface Ni-O peak (yellow)^31, 32^. After repeated OER cycles, a new main peak (violet) assigned to Ni-OH appeared^33, 34^. To clarify the oxidation state of Ni, the valence state was examined by linear combination fitting method assuming the cycled Ni_2_P samples include Ni_2_P and NiO components (inset in Fig. 6a). The valence states of as-prepared Ni_2_P and NiO were set to be +1.5 and +2. The XANES spectrum of 5 cycle was composed of 42% Ni_2_P and 58% NiO while the spectrum of 1000 cycle was composed of 100% NiO, which gives +1.8 and +2 valence states for 5 cycle and 1000 cycle, respectively. Similarly, the reduced FFT TEM patterns corresponding to Ni(OH)_2_(101) and NiO(200) show nickel phosphide oxidation. Microstructural analysis using extended X-ray fine structure (EXAFS) analysis of the Ni *K* edge (Fig. 6c) reinforced these observations; after deformation, the FFT signal changed completely, with Ni-O and Ni-Ni peaks developing in a manner consistent with the Ni(OH)_2_ phase^31, 32^. After five cycles, the Ni-P signal dropped dramatically and almost diminished through 3,000 CV scans (Supplementary Fig. S5). We carefully suggested that P species should have a role to promote deformation of Ni_2_P and formation of the film-like oxidized Ni species. Again, this indicates that the surface P species dissolved into the solution through consecutive cycles. In addition, the crystallinity of Ni-O and Ni-Ni was enhanced, as observed by the increasing FFT magnitude. Previous research has shown that *α*-Ni(OH)_2_ spontaneously converts to *β*-Ni(OH)_2_ under alkaline conditions^23^, particularly in electrochemical potential cycles^33–36^. During this process, the Ni(OH)_2_ crystallinity improves as stacking faults in the oxy-hydroxides disappear^34, 37–39^. According to the reported transformation of Ni(OH)_2_ and our observed EXAFS signals, the Ni_2_P/C structural changes must also involve the transformation to a highly ordered *β*-Ni(OH)_2_ phase. Furthermore, this transformation did not occur in NiO/C, as shown in Supplementary Fig. S6, indicating that Ni_2_P/C is a unique precursor for OER catalysis. More detailed discussions concerning these changes encompassing the improved OER activity of Ni-based electrocatalysts are given below.

Interestingly, the reduction peaks of the reacted Ni_2_P/C surfaces, which are associated with Ni(OH)_2_ + *n*e^−^ → NiOOH, are clearly separated into two peaks at 1.27 and 1.37 V_RHE_ after consecutive CV scans (Fig. 5a). The reduction characteristics continued to decrease after 500 CV scans, occurring at the same time as the discussed structural and chemical transformations (inset in Fig. 5b). Furthermore, the area of the two peaks exhibited an inverse trend after repeated scans (Fig. 5c), with the peak at 1.27 V decreasing and the one at 1.37 V increasing up to 6,000 scans. However, the ratio between the two peaks approached approximately 0.3. The OER activity also reached a maximum at 6,000 CV scans (Fig. 3e), suggesting that the anomalous activation and *in situ* structural changes of Ni_2_P/C are introduced during the electrochemical redox reactions.

**Figure 7:**
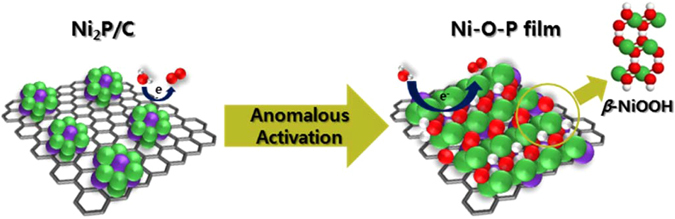
Schematic diagram of the anomalous *in situ* transformation of Ni_2_P to *β*-NiOOH for a robust OER catalyst.

The changes of the NiOOH phase affect its catalytic activity at the potential range of the OER. NiOOH can exist as either the *β*- or *γ*-phase in an alkaline aqueous solution^40–43^. Subbaraman *et al*. demonstrated that the OER activity of M-OOH (M = Ni, Co, Fe, or Mn) in alkaline solution is determined solely by the energetics of the OH-MOOH species^25^. An interaction that is too strong might slow the OER rate because of the excessive stabilization of the reaction intermediates. Our calculations clearly show that the binding energy of OH (*E*_B_(OH)) for *β*-NiOOH (4.90 eV) was relatively small compared to that of *γ*-NiOOH (1.57 eV) (Fig. 5c). Likewise, the two different peaks at 1.37 and 1.27 V_RHE_ correspond to the *β*- and *γ*- phase of NiOOH, respectively^40–43^. As shown in Fig. 6b, the *β*-phase portion steadily increases and saturates through 6,000 CV scans and is accompanied by an improved OER performance and a drastic reduction in peaks assigned to *γ*-NiOOH. Recall that we predicted increases of the *β*-Ni(OH)_2_ phase from EXAFS signals, suggesting that formation of *β*-NiOOH is favourable according to Fig. 1. Although some studies have demonstrated that the NiP_*x*_@NiO_*y*_ core-shell structure formed during electrocatalysis can enhance OER activity^11–13, 15^, the source of this superior activity is not yet known. Herein, the results of physicochemical characterizations imply that the anomalous activation behaviours of Ni_2_P/C electrocatalysts towards the OER are derived from the facile formation of nickel phosphide to *β*-NiOOH with a high surface area in repeated CV scans, probably by the aid of underlying P (Fig. 7). Therefore, this spontaneous phase transformation during the OER circumstance leads to such a high activity and stability.

In summary, Ni_2_P/C was synthesized and subjected to electrochemical testing, ultimately forming a robust OER electrocatalyst because of the structural transformation. This enhanced performance resulted from the formation of a Ni-O-P film-like structure with a high surface area that was layered onto the carbon support and the preferential growth of *β*-NiOOH, providing highly active sites for OER. This favourable transition to the *β*-phase proceeds up to 6,000 CV scans, resulting in continuous enhancement of the OER. After completing this transformation, the outstanding OER activity is stably maintained. These results offer new methods for the initial preparation of OER electrocatalysts that provide excellent catalytic performances.

## Methods

### Synthesis of the Electrocatalysts

Carbon-supported Ni_2_P nanoparticles were prepared by thermal decomposition following a previously reported method^31^. First, nickel(II) acetylacetonate (Ni(acac)_2_) and (CH_3_(CH_2_)_7_)_3_P were mixed and stirred at 90 °C in an inert atmosphere. Then, the solution was quickly added to (CH_3_(CH_2_)_7_)_3_PO, heated to 310 °C, and stirred for 2 h. After the precipitates formed, the reactor was cooled to room temperature and the Ni_2_P nanoparticles were collected. The Ni(acac)_2_:(CH_3_(CH_2_)_7_)_3_P:(CH_3_(CH_2_)_7_)_3_PO molar ratio was 1:15:20. To acquire 50 wt% Ni_2_P/C, the nanoparticles were mixed with Vulcan XC-72 carbon black in CH_3_CON(CH_3_)_2_ using a wand-sonicator for 1 h. Then, the prepared catalyst was washed several times and used without further purification. For comparison, carbon-supported NiO nanoparticles were prepared by simple mixing of NiO nanoparticles (<50 nm) and carbon black. The typical preparation method was as follows: NiO nanoparticles and carbon black were sonicated in dimethylacetamide for 1 h. Then, the precipitates were filtered and washed in ethanol several times. The collected powder was dried in a vacuum oven at 60 °C. These particles were denoted as NiO/C.

### Electrochemical Measurements

To evaluate the electrochemical properties, we performed half-cell measurements using a three-electrode system, consisting of a working electrode (glassy carbon electrode, diameter = 5 mm), reference electrode (saturated calomel electrode), and counter electrode (Pt wire). The catalyst ink was prepared by mixing Ni_2_P/C, NiO/C, or IrO_x_/C (10 mg), a Nafion perfluorinated resin solution (57.2 μL), and CH_3_CON(CH_3_)_2_ (1.6 mL). The ink was transferred onto a rotating glassy carbon electrode (diameter = 5 mm). The catalyst loading was set at 0.15 mg cm^−2^. CV (1.0 to 1.8 V_RHE_) was used to measure the OER at a scan rate of 5 mV s^−1^ in 0.1 M KOH with saturated O_2_ at room temperature. For consecutive CV scans, the applied potentials were swept from 1.0 to 1.8 V_RHE_ at a faster scan rate of 100 mV s^−1^. During oxygen evolution, the working electrode was rotated with a rotation speed of 1,600 rpm to remove the oxygen bubbles on the surface. All measurements were standardized vs. RHE and were compensated for the solution resistance, which was obtained from electrochemical impedance spectroscopy. The frequency range was 0.5–200 kHz with an amplitude of 0.025 V. Commercial IrO_x_/C and NiO/C were used as reference catalysts for comparisons.

### Characterization

We used various physicochemical techniques to perform *ex situ* characterizations of the Ni-P-O electrocatalyst. HR-TEM images were obtained using a Titan 80–300 microscope (FEI) with an acceleration voltage of 300 kV. Spherical aberration (C_s_)–corrected STEM images were also acquired using the same equipment. Elemental analysis was conducted by EDS using a Titan 80–300 microscope. Crystallographic information was obtained from XRD patterns (D/Max 2500/PC, Rigaku). The operating conditions were 40 kV and 200 mA with a scan range (2*θ*) between 30° and 80° at 0.5 ° min^−1^. X-ray absorption spectroscopy was performed using the 10 C beamline at the Pohang Accelerator Laboratory (PAL). The Ni *K* edge was collected in fluorescence mode at room temperature. The absorption edge of Ni *K* (*E*_0_ = 8,333 eV) was calibrated using Ni foil. X-ray absorption near-edge structure (XANES) and extended X-ray absorption fine structure (EXAFS) were analysed by the IFEFFIT software package using ATHENA and ARITEMIS. The experimental details were previously described^44^. X-ray photoelectron spectroscopy (XPS) was performed using a Theta Probe AR-XPS system (Thermo Fisher Scientific) to compare the surface compositions of the nanoparticles. Monochromatic Al *K*_α_ (1486.6 eV) radiation was used as an X-ray source. All spectra were obtained at 15 kV and 150 W with a spot size of 400 μm and calibrated with respect to the C 1 *s* peak at 284.6 eV.

## Electronic supplementary material

SUPPLEMENTARY INFORMATION

## References

[1] Walter MG (2010). Solar Water Splitting Cells. Chem. Rev..

[2] Cook TR (2010). Solar Energy Supply and Storage for the Legacy and Nonlegacy Worlds. Chem. Rev..

[3] McCrory CCL (2015). Benchmarking Hydrogen Evolving Reaction and Oxygen Evolving Reaction Electrocatalysts for Solar Water Splitting Devices. J. Am. Chem. Soc..

[4] Man IC (2011). Universality in oxygen evolution electrocatalysis on oxide surfaces. ChemCatChem.

[5] McCrory CCL, Jung S, Peters JC, Jaramillo TF (2013). Benchmarking Heterogeneous Electrocatalysts for the Oxygen Evolution Reaction. J. Am. Chem. Soc..

[6] Ren, J., Antonietti, M. & Fellinger, T. P. Efficient Water Splitting Using a Simple Ni/N/C Paper Electrocatalyst. *Adv. Energy Mater*. **5** (2015).

[7] Nardi, K. L., Yang, N., Dickens, C. F., Strickler, A. L. & Bent, S. F. Creating Highly Active Atomic Layer Deposited NiO Electrocatalysts for the Oxygen Evolution Reaction. *Adv. Energy Mater*. **5** (2015).

[8] Bediako DK (2012). Structure–Activity Correlations in a Nickel–Borate Oxygen Evolution Catalyst. J. Am. Chem. Soc..

[9] Zhao Y, Nakamura R, Kamiya K, Nakanishi S, Hashimoto K (2013). Nitrogen-doped carbon nanomaterials as non-metal electrocatalysts for water oxidation. Nat. Commun..

[10] Stern L-A, Hu X (2014). Enhanced oxygen evolution activity by NiO_x_ and Ni(OH)_2_ nanoparticles. Faraday Discuss..

[11] Ledendecker M (2015). The Synthesis of Nanostructured Ni_5_P_4_ Films and their Use as a Non‐Noble Bifunctional Electrocatalyst for Full Water Splitting. Angew. Chem. Int. Ed..

[12] Stern L-A, Feng L, Song F, Hu X (2015). Ni_2_P as a Janus catalyst for water splitting: the oxygen evolution activity of Ni_2_P nanoparticles. Energy Environ. Sci..

[13] Han A, Chen H, Sun Z, Xu J, Du P (2015). High catalytic activity for water oxidation based on nanostructured nickel phosphide precursors. Chem. Commun..

[14] Li Z, Dou X, Zhao Y, Wu C (2016). Enhanced oxygen evolution reaction of metallic nickel phosphide nanosheets by surface modification. Inorg. Chem. Front..

[15] Yu X-Y, Feng Y, Guan B, Lou XW, Paik U (2016). Carbon coated porous nickel phosphides nanoplates for highly efficient oxygen evolution reaction. Energy Environ. Sci..

[16] Zhao Y (2015). Graphene-Co_3_O_4_ nanocomposite as electrocatalyst with high performance for oxygen evolution reaction. Sci. Rep..

[17] Zhuang Z, Sheng W, Yan Y (2014). Synthesis of Monodispere Au@Co_3_O_4_ Core‐Shell Nanocrystals and Their Enhanced Catalytic Activity for Oxygen Evolution Reaction. Adv. Mater..

[18] Masa J (2014). Mn_x_O_y_/NC and Co_x_O_y_/NC Nanoparticles Embedded in a Nitrogen‐Doped Carbon Matrix for High‐Performance Bifunctional Oxygen Electrodes. Angew. Chem. Int. Ed..

[19] Kanan MW, Nocera DG (2008). In situ formation of an oxygen-evolving catalyst in neutral water containing phosphate and Co^2+^. Science.

[20] Friebel D (2015). Identification of highly active Fe sites in (Ni, Fe) OOH for electrocatalytic water splitting. J. Am. Chem. Soc..

[21] Louie MW, Bell AT (2013). An Investigation of Thin-Film Ni–Fe Oxide Catalysts for the Electrochemical Evolution of Oxygen. J. Am. Chem. Soc..

[22] Qian, L. *et al*. Trinary Layered Double Hydroxides as High‐Performance Bifunctional Materials for Oxygen Electrocatalysis. *Adv. Energy Mater*. **5** (2015).

[23] Bode H, Dehmelt K, Witte J (1966). Zur kenntnis der nickelhydroxidelektrode—I. Über das nickel (II)-hydroxidhydrat. Electrochim. Acta.

[24] Van der Ven A, Morgan D, Meng Y, Ceder G (2006). Phase stability of nickel hydroxides and oxyhydroxides. J. Electrochem. Soc..

[25] Subbaraman R (2012). Trends in activity for the water electrolyser reactions on 3d M(Ni,Co,Fe,Mn) hydr(oxy)oxide catalysts. Nat. Mater..

[26] Li Y-F, Selloni A (2014). Mechanism and Activity of Water Oxidation on Selected Surfaces of Pure and Fe-Doped NiOx. ACS Catal..

[27] Ma TY, Dai S, Jaroniec M, Qiao SZ (2014). Graphitic Carbon Nitride Nanosheet–Carbon Nanotube Three‐Dimensional Porous Composites as High‐Performance Oxygen Evolution Electrocatalysts. Angew. Chem. Int. Ed..

[28] Gao M-R (2014). Nitrogen-Doped Graphene Supported CoSe_2_ Nanobelt Composite Catalyst for Efficient Water Oxidation. ACS Nano.

[29] Gao M (2014). Efficient water oxidation using nanostructured *α*-nickel-hydroxide as an electrocatalyst. J. Am. Chem. Soc..

[30] Trotochaud L, Young SL, Ranney JK, Boettcher SW (2014). Nickel–Iron Oxyhydroxide Oxygen-Evolution Electrocatalysts: The Role of Intentional and Incidental Iron Incorporation. J. Am. Chem. Soc..

[31] Seo H-R, Cho K-S, Lee Y-K (2011). Formation mechanisms of Ni_2_P nanocrystals using XANES and EXAFS spectroscopy. Mater. Sci. Eng., B.

[32] Zhu Y (2014). Ultrathin Nickel Hydroxide and Oxide Nanosheets: Synthesis, Characterizations and Excellent Supercapacitor Performances. Sci. Rep..

[33] Kamath PV (1994). Stabilized α‐Ni(OH)_2_ as Electrode Material for Alkaline Secondary Cells. J. Electrochem. Soc..

[34] Tessier C (2000). Structural and textural evolution of zinc-substituted nickel hydroxide electrode materials upon ageing in KOH and upon redox cycling. Solid State Ionics.

[35] Godwin I, Lyons M (2013). Enhanced oxygen evolution at hydrous nickel oxide electrodes via electrochemical ageing in alkaline solution. Electrochem. Commun..

[36] Desilvestro J, Corrigan DA, Weaver MJ (1988). Characterization of redox states of nickel hydroxide film electrodes by *in situ* surface Raman spectroscopy. J. Electrochem. Soc..

[37] Ramesh T, Kamath PV (2006). Synthesis of nickel hydroxide: effect of precipitation conditions on phase selectivity and structural disorder. J. Power Sources.

[38] Deabate S, Fourgeot F, Henn F (2000). X-ray diffraction and micro-Raman spectroscopy analysis of new nickel hydroxide obtained by electrodialysis. J. Power Sources.

[39] Barde F, Palacin M, Chabre Y, Isnard O, Tarascon J-M (2004). *In situ* neutron powder diffraction of a nickel hydroxide electrode. Chem. Mater..

[40] Hall DS, Bock C, MacDougall BR (2013). The electrochemistry of metallic nickel: oxides, hydroxides, hydrides and alkaline hydrogen evolution. J. Electrochem. Soc..

[41] Marioli JM, Sereno LE (1995). The potentiodynamic behavior of nickel-chromium (80: 20) alloy electrodes in 0.10 N sodium hydroxide. Electrochim. Acta.

[42] Barnard R, Randell C, Tye F (1980). Studies concerning charged nickel hydroxide electrodes I. Measurement of reversible potentials. J. Appl. Electrochem..

[43] Jović B, Lačnjevac U, Jović V, Krstajić N (2015). Kinetics of the oxygen evolution reaction on NiSn electrodes in alkaline solutions. J. Electroanal. Chem..

[44] Yoo SJ (2011). Enhanced stability and activity of Pt-Y alloy catalysts for electrocatalytic oxygen reduction. Chem. Commun..

