# Supplementary material for: Anomalous in situ Activation of Carbon-Supported Ni2P Nanoparticles for Oxygen Evolving Electrocatalysis in Alkaline Media
Source: Sci Rep. 2017 Aug 15;7:8236. doi: 10.1038/s41598-017-08296-0 (PMC5557805; doi:10.1038/s41598-017-08296-0)
Supplement: Supplementary file 1 — SUPPLEMENTARY INFORMATION [file 41598_2017_8296_MOESM1_ESM.pdf]

Supporting Information for

Anomalous *in-situ* Activation of Carbon Supported Ni_2P Nanoparticles for the Oxygen Evolving Electrocatalysis in Alkaline Media

Young-Hoon Chung,[1,2,†] Injoon Jang,[1,5,†] Jue-Hyuk Jang,[1,3] Hyun S. Park,[1] Hyung Chul Ham,[1] Jong Hyun Jang,[1,3] Yong-Kul Lee,[4] Sung Jong Yoo[1★]

[1] *Fuel Cell Research Center, Korea Institute of Science and Technology (KIST), 02792 Seoul, Republic of Korea*

[2] *Applied Materials Examination Division, Korean Intellectual Property Office (KIPO), 35208 Daejeon, Republic of Korea*

[3] *Green School, Korea University, 02841 Seoul, Republic of Korea*

[4] *Department of Chemical Engineering, Dankook University, 16890 Yongin, Republic of Korea*

[5] *School of Chemical and Biological Engineering, Seoul National University (SNU), Seoul 08826, Republic of Korea*

[†] These authors contributed equally to this work.

[★] Corresponding author e-mail: ysj@kist.re.kr

Contents:

1. **Experimental Details**
   1.1. Chemicals
   1.2. KOH Purification
   1.3. Computational Methods

2. **Supplementary Data**
   2.1. Oxygen Evolution Reaction (OER) Measurements
   2.2. Scanning Transmission Electron Microscope (STEM) Analysis
   2.3. X-ray Photoelectron Spectroscopy (XPS) Spectra
   2.4. X-ray Absorption Fine Structure (XAFS) Analysis
   2.5. *In-situ* Structural Information
   2.6. Density Functional Theory (DFT) Calculations
   2.7. X-ray Photoelectron Spectroscopy (XPS) Data
   2.8. Literature Survey

3. **References**

1. Experimental Details

1.1. Chemicals

Nickel(II) acetylacetonate ($Ni(acac)_2$, $C_{10}H_{14}NiO_4$, 95%) and tri-octylphosphine (TOP, $(CH_3(CH_2)_7)_3P$, 95%) were received from Alfa-Aesar®. Nickel(II) nitrate hexahydrate ($Ni(NO_3)_2{\cdot}6H_2O$, 99.999%), tri-octylphosphine oxide (TOPO, $(CH_3(CH_2)_7)_3P$, 90%), N,N-dimethylacetamide (DMAc, $CH_3CON(CH_3)_2$, 99.5%), nickel oxide nanoparticle (NiO, < 50 mn)and Nafion® perfluorinated resin solution (5 wt%) are purchased from Sigma-Aldrich®. Potassium hydroxide (KOH) was purchased by Junsei Chemical Co. Ltd. Carbon black (Vulcan XC-72, Carbot corp.) and IrO_x/C (20 wt%, Johnson-Matthey) was received.

1.2. KOH Purification

In order to remove trace amount of the Fe ions, we carried out KOH solution purification step using $Ni(OH)_2$, as reported previously[1]. Typical procedure is as follows: ($Ni(NO_3)_2{\cdot}6H_2O$ (2 g, dissolved in 4 mL of H_2O) was added into the 1 M KOH solution (20 mL). The mixture was vigorously stirred until the fully formation of green precipitates, $Ni(OH)_2$ and supernatant was decanted for several times. Then, obtained $Ni(OH)_2$ was redispersed 0.1 M KOH (50 mL) for purification. The mixture was stirred and stored overnight. The purified 0.1 M KOH was used for the OER measurements without trace level of Fe ions.

1.3. Computational Methods

The calculations reported herein were performed on the basis of spin polarized density functional theory (DFT) within the generalized gradient approximation (GGA-PBE)[2], as implemented in the Vienna Ab-initio Simulation Package (VASP)[3]. The projector augmented wave (PAW) method with a planewave basis set was employed to describe the interaction between core and valence electrons[4]. An energy cutoff of 400 eV was applied for the planewave expansion of the electronic eigenfunctions. For the Brillouin zone integration, we used a (4×4×1) Monkhorst-Pack mesh of k points to calculate geometries and total energies[5]. For better describing the localization of electrons in nickel hydroxides and oxyhydroxides, we applied the Hubbard U-J correction [U-J =6.4 for Ni][6], as implemented in Dudarev *et al*[7].

For *β*-NiOOH(100) model surface (See Figure S12), we constructed a 2×2 slab consisting of a eight atomic layers terminated by OH (the crystal structure of β-NiOOH has O_A-O_A-Ni-O_B-O_B-Ni-O_C-O_C stacking sequence (AABBCC))[8]. For the α γ-NiOOH(100) surface we put one H_2O molecule in the interlayer of β-NiOOH(100) (that is, we inserted one H_2O and Na into the interlayer of β-NiOOH(100) (O_A--H_2O--O_A-Ni-O_B--Na--O_B-Ni-O_C-O_C)). The slab is separated from its periodic images in the vertical direction by a vacuum space corresponding to ~15Å. While the bottom three layers of the eight-layer slab were fixed at corresponding bulk positions, the upper five layers were fully relaxed using the conjugate gradient method until residual forces on all the constituent atoms become smaller than 5×10^{-2} eV/Å .

The OH binding energies for β-NiOOH(100) and γ-NiOOH(100) surfaces are calculated using the following equation.

E_{OH}= E[OH vacancy]-E[OH]- E[nickel oxyhydroxides]

Where, E[nickel hydroxides or oxyhydroxides] is the total energy of β-NiOOH(100) and γ-NiOOH(100), E[OH vacancy] is the total energy of β-NiOOH(100) and γ-NiOOH(100) with an OH vacancy, E[OH] is the total energy of OH in gas phase.

2. Supplementary Data

2.1. Oxygen Evolution Reaction (OER) Measurements

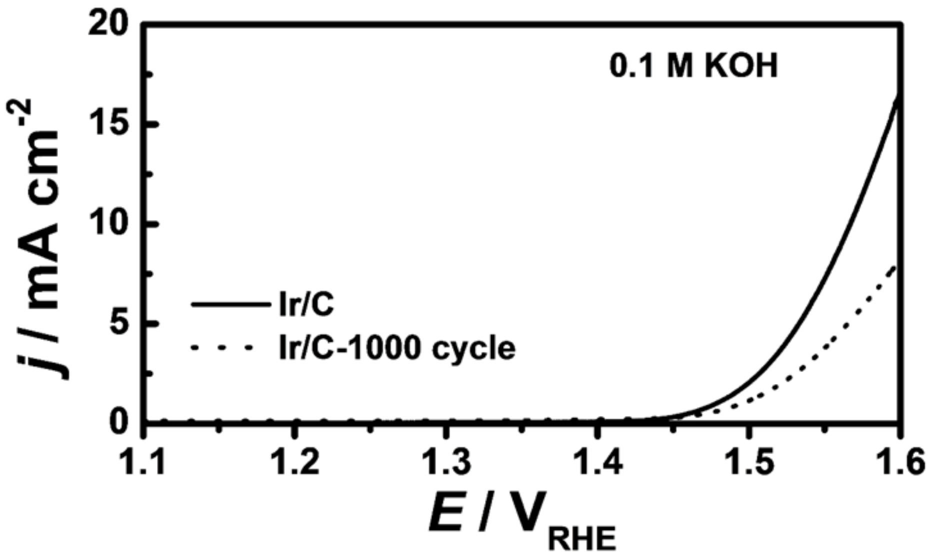

Figure S1. Accelerated durable test (ADT) of Ir/C electrocatalyst after 1000 potential cycling measurements.

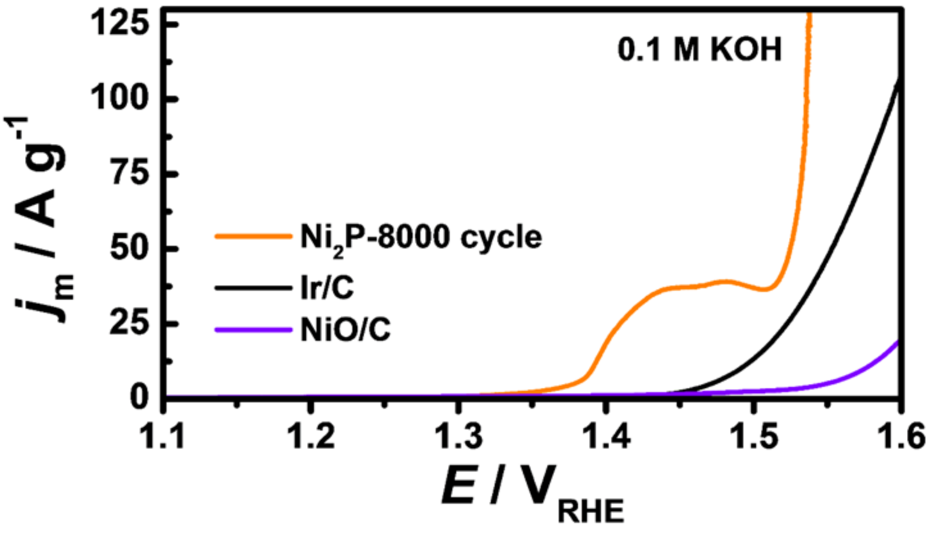

Figure S2. Polarization curves of oxygen evolution reaction (OER) using mass-normalized current density (J_m) based on loaded electrocatalysts on glassy carbon electrode (0.15 mg cm^{-2}).

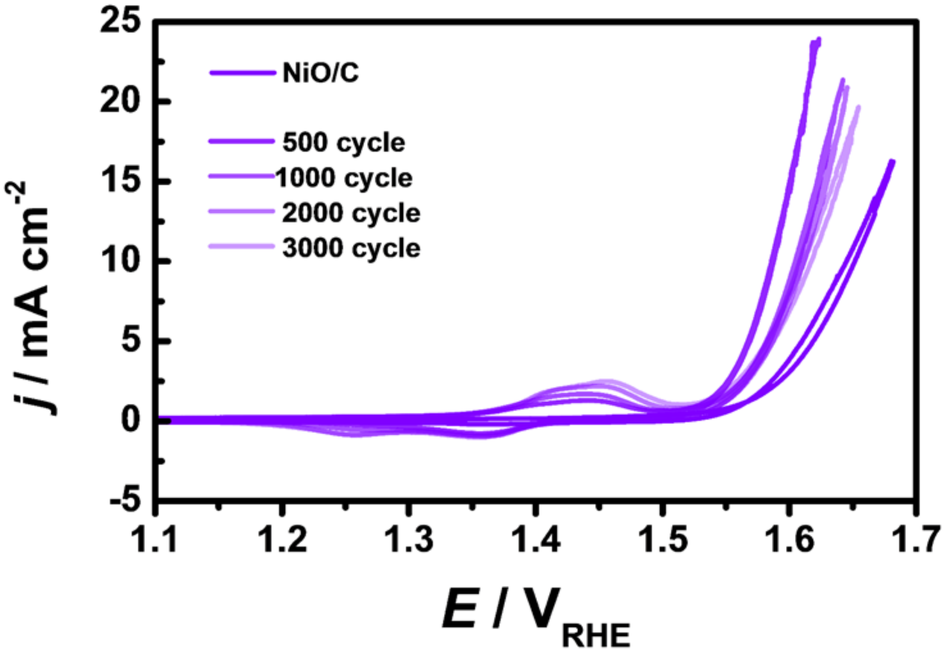

Figure S3. Polarization curves of oxygen evolution reaction (OER) for NiO/C in 0.1 M KOH with respect to the potential cycles.

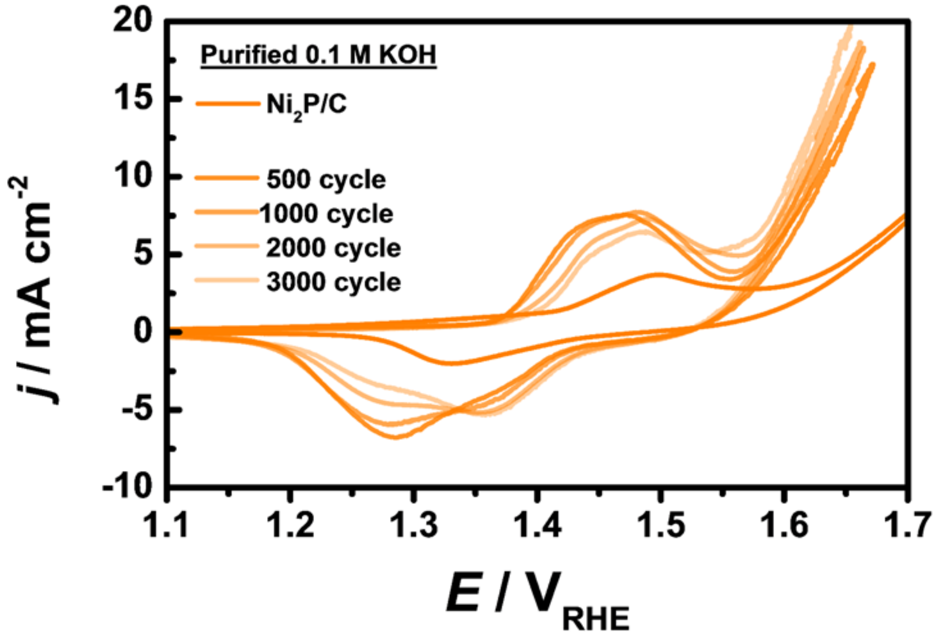

Figure S4. Polarization curves of oxygen evolution reaction (OER) for Ni_2P/C in 0.1 M KOH in the absence of trace amount of Fe ions with respect to the potential cycles.

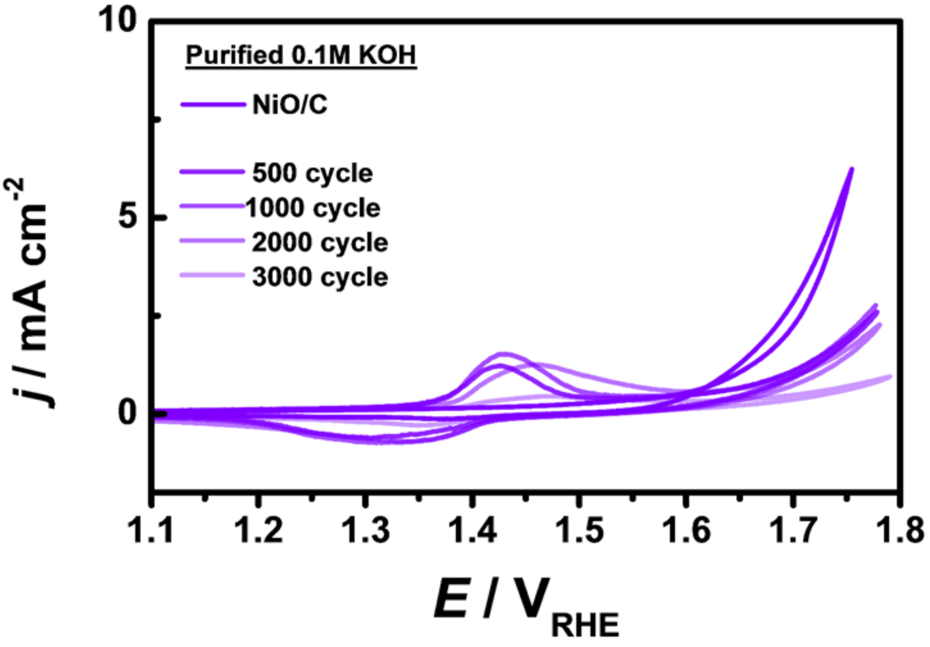

Figure S5. Polarization curves of oxygen evolution reaction (OER) for NiO/C in 0.1 M KOH in the absence of trace amount of Fe ions with respect to the potential cycles.

2.2. Scanning Transmission Electron Microscope (STEM) Analysis

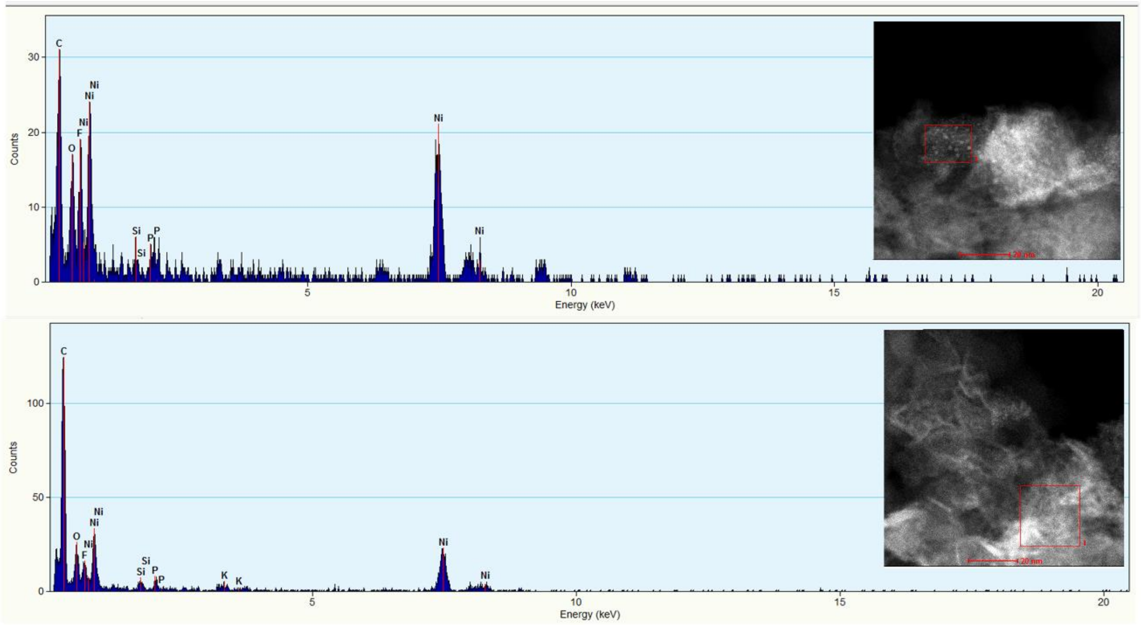

Figure S6. High angle annular dark field (HADDF) images and energy dispersive X-ray spectroscopy (EDS) spectra of Ni_2P/C after the oxygen evolution reaction (OER).

2.3. Photoelectron Spectroscopy (XPS) Spectra

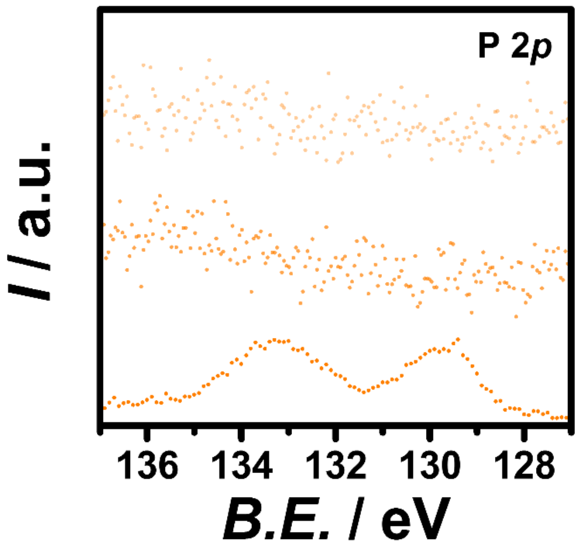

Figure S7. X-ray photoelectron spectroscopy (XPS) spectra of P 2p: as-prepared Ni_2P (bottom), 5 cycle (middle), and 1000 cycle (top) after the oxygen evolution reaction (OER).

2.4. X-ray Absorption Fine Structure (XAFS) Analysis

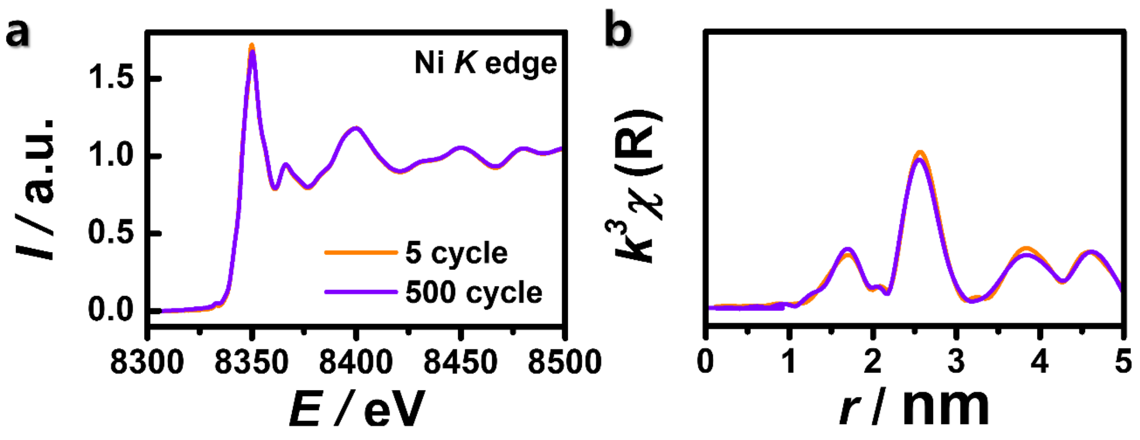

Figure S8. (a) X-ray absorption near-edge structure (XANES) and (b) extended X-ray fine structure (EXAFS) of Ni *K* edge (E_0 = 8333 eV) of NiO/C after oxygen evolution reaction (OER).

2.5. *In-situ* Structural Information

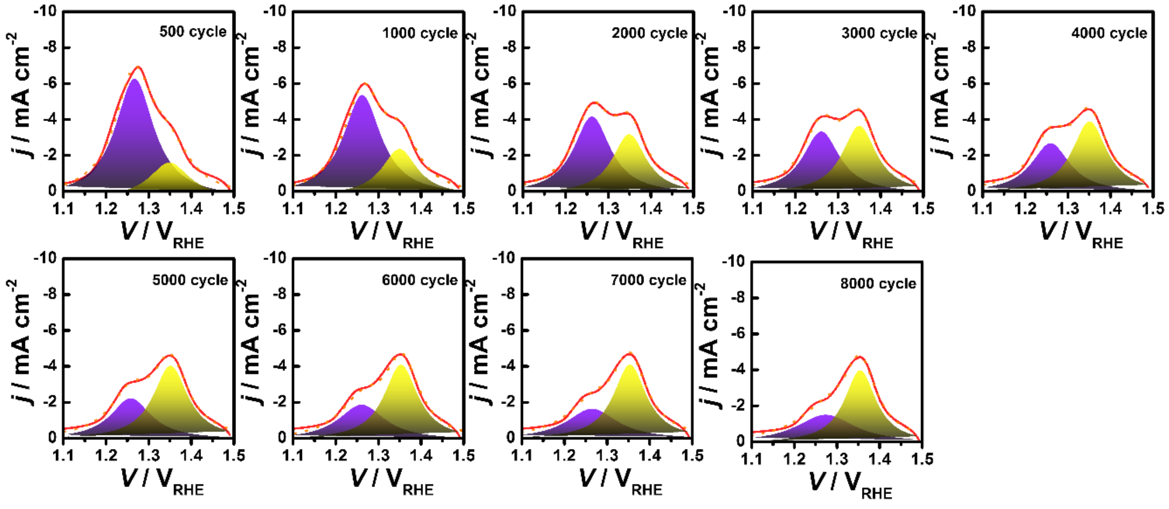

Figure S9. Deconvolution of reduction peaks of NiOOH to $Ni(OH)_2$ with respect to the potential cycles .

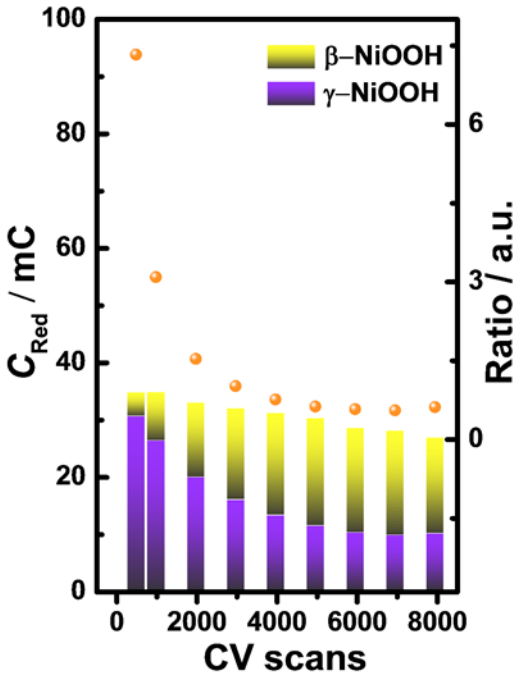

Figure S10. Charges assigned to the reduction current related to β-NiOOH and γ-NiOOH phase estimated by the deconvolution reduction peaks and absolute ration of γ-NiOOH to β-NiOOH phase in in repeated cyclic voltamograms (CVs) of Ni-P-O electrocatalysts.

2.6. Density Functional Theory (DFT) Calculation

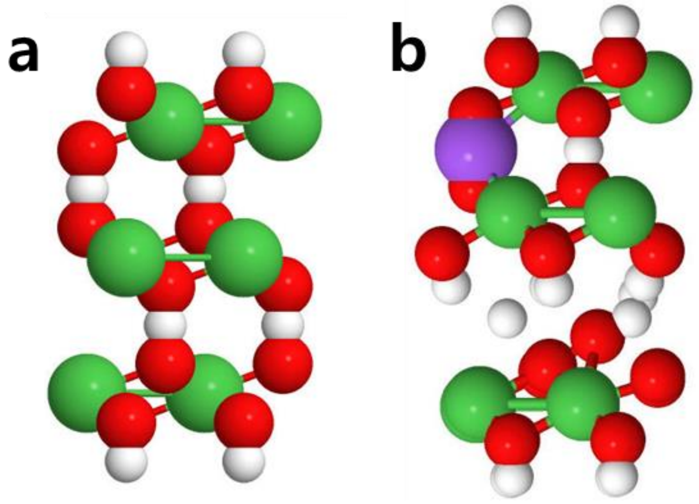

Figure S11. Side views of model (100) surfaces; (a) β-NiOOH and (b) γ-NiOOH. The red, green, white and purple spheres indicate O, Ni, H and Na atoms, respectively

2.7. X-ray Photoelectron Spectroscopy (XPS) Data

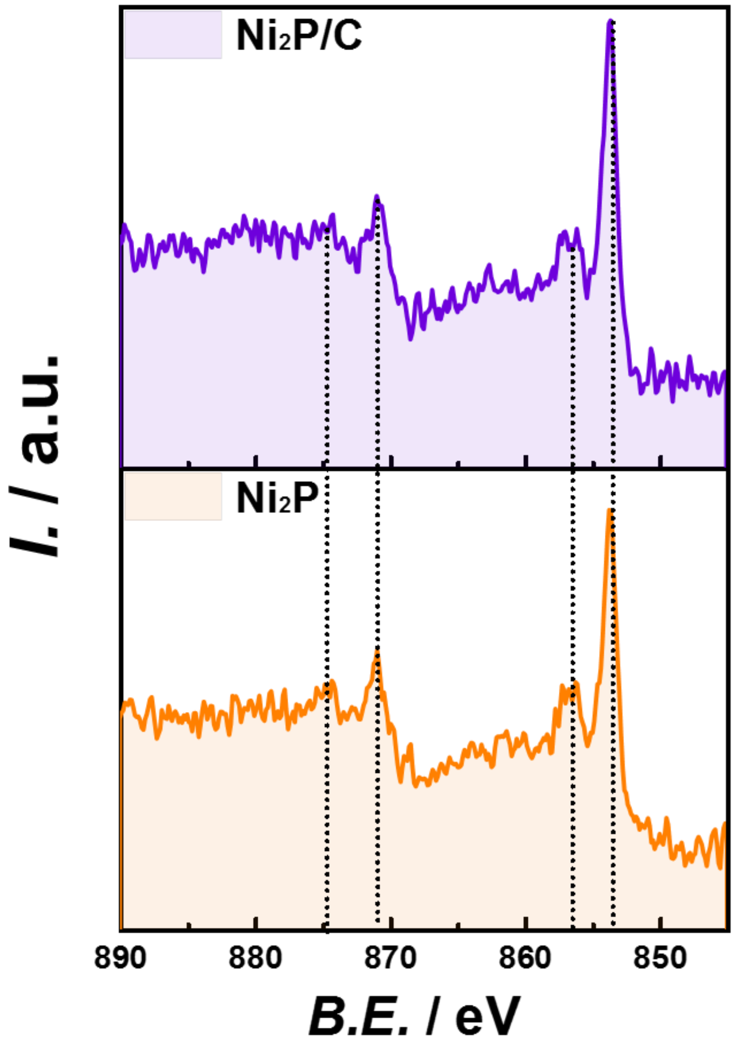

Figure S12. X-ray photoelectron spectroscopy (XPS) of Ni 2*p* states for Ni_2P and Ni_2P/C

2.8 Literature Survey

Table S1. Overpotentials of the oxygen evolution reaction (OER) at 10 mA cm^{-2} in 0.1 M KOH for the state-of-the-art electrocatalysts[9-19].

Electrocatalysts	Overpotential (mV) @10 mA cm^{-2}	Loading (mg cm^{-2})	Reference
**Ni_2P/C**	**300**	**0.15**	**Our work**
rGO@CoNiOx	320	0.2	[9]
Co@NS/CNTsMCF-900	357	0.6	[10]
N-Co_9S_8/graphene	409	0.2	[11]
CoNP@NC/NG-700	390	N/D	[12]
Ni_2Co_1/Ni_2Co_1Ox	380	0.4	[13]
$0.61NiMnO_3/NiMn_2O_4$	380	0.24	[14]
$NiCo_2O_4$/rGO-NMP	450	0.24	[15]
$NiFe_2O_4$/α-$Ni(OH)_2$	341	0.2	[16]
Graphene-Co_3O_4	359	0.19	[17]
γ-$Ni_{0.75}Fe_{0.25}OOH$	370	N/D	[18]
Oxidative NiCoFe-LDH	340	0.12	[19]
Co_xO_y/NC	430	0.21	[20]
3D g-C_3N_4 NS-CNT	370	0.20	[21]
Au@Co_3O_4	390	0.06	[22]
Ni@NC	390	0.4	[23]
N-doped graphene $CoSe_2$	366	0.2	[24]
N doped C-NiO_x	420	0.2	[25]
α-$Ni(OH)_2$ sphere	331	0.2	[26]
Nickel phosphide nanosheet	347	0.285	[27]

3. References

1 Trotochaud, L., Young, S. L., Ranney, J. K. & Boettcher, S. W. Nickel–iron oxyhydroxide oxygen-evolution electrocatalysts: The role of intentional and incidental iron incorporation. *J. Am. Chem. Soc.* **136**, 6744-6753, (2014).

2 Perdew, J. P., Burke, K. & Ernzerhof, M. Generalized Gradient Approximation Made Simple. *Phys. Rev. Lett.* **77**, 3865-3868, (1996).

3 Kresse, G. & Furthmüller, J. Efficient iterative schemes for *ab initio* total-energy calculations using a plane-wave basis set. *Phys. Rev. B* **54**, 11169-11186, (1996).

4 Blöchl, P. E. Projector augmented-wave method. *Phys. Rev. B* **50**, 17953-17979, (1994).

5 Monkhorst, H. J. & Pack, J. D. Special points for Brillouin-zone integrations. *Phys. Rev. B* **13**, 5188-5192, (1976).

6 Wang, L., Maxisch, T. & Ceder, G. Oxidation energies of transition metal oxides within the GGA+U framework. *Phys. Rev. B* **73**, 195107, (2006).

7 Dudarev, S. L., Botton, G. A., Savrasov, S. Y., Humphreys, C. J. & Sutton, A. P. Electron-energy-loss spectra and the structural stability of nickel oxide: An LSDA+U study. *Phys. Rev. B* **57**, 1505-1509, (1998).

8 Van der Ven, A., Morgan, D., Meng, Y. & Ceder, G. Phase stability of nickel hydroxides and oxyhydroxides. *J. Electrochem. Soc.* **153**, A210-A215, (2006).

9 Ping Li & Hua Chun Zeng, Sandwich-Like Nanocomposite of CoNiOx/Reduced Graphene Oxide for Enhanced Electrocatalytic Water Oxidation, *Adv. Funct. Mater.*, **27**, 1606325, (2017)

10 Z. Wang *et al.*, Cobalt nanoparticles encapsulated in carbon nanotube-grafted nitrogen and sulfur co-doped multichannel carbon fibers as efficient bifunctional oxygen electrocatalysts, *J. Mater. Chem. A*, **5,** 4949-4961, (2017)

11 Shuo Dou, Li Tao, Jia Huo, Shuangyin Wang & Liming Dai, Etched and doped Co_9S_8/graphene hybrid for oxygen electrocatalysis, *Energy Environ. Sci.*, **9**, 1320, (2016)

12 X. Zhong *et al*. Integrating cobalt phosphide and cobalt nitrideembedded nitrogen-rich nanocarbons: highperformance bifunctional electrocatalysts for oxygen reduction and evolution, , *J. Mater. Chem. A*, **4,** 10575-10584, (2016)

13 Jinling He , Binbin Hu & Yong Zhao, Superaerophobic Electrode with Metal@Metal-Oxide Powder Catalyst for Oxygen Evolution Reaction, *Adv. Funct. Mater.*, **26**, 5998–6004, (2016)

14 X. He *et al.*, $NiMnO_3$/$NiMn_2O_4$ Oxides Synthesized via the Aid of Pollen: Ilmenite/Spinel Hybrid Nanoparticles for Highly Efficient Bifunctional Oxygen Electrocatalysis, *ACS Appl. Mater. Interfaces*, **8**, 26740−26757, (2016)

15 Ediga Umeshbabu, G. Rajeshkhanna, Ponniah Justin & G. Ranga Rao, $NiCo_2O_4$/rGO hybrid nanostructures for efficient electrocatalytic oxygen evolution, *J Solid State Electrochem*, **20**, 2725–2736, (2016)

16 H. Chen *et al.*, One-pot fabrication of $NiFe_2O_4$ nanoparticles on a-$Ni(OH)_2$ nanosheet for enhanced water oxidation, *J. Power Sources*, **324**, 499-508, (2016)

17 Zhao, Y. *et al.* Graphene-Co_3O_4 nanocomposite as electrocatalyst with high performance for oxygen evolution reaction. *Sci. Rep.* **5**, 7629 (2015).

18 Friebel, D. *et al.* Identification of highly active Fe sites in (Ni, Fe) OOH for electrocatalytic water splitting. *J. Am. Chem. Soc.* **137**, 1305-1313, (2015).

19 Qian, L. *et al.* Trinary Layered Double Hydroxides as High-Performance Bifunctional Materials for Oxygen Electrocatalysis. *Adv. Energy Mater.* **5**, (2015).

20 Masa, J. *et al.* Mn_xO_y/NC and Co_xO_y/NC Nanoparticles Embedded in a Nitrogen-Doped Carbon Matrix for High-Performance Bifunctional Oxygen Electrodes. *Angew. Chem. Int. Ed.* **53**, 8508-8512, (2014).

21 Ma, T. Y., Dai, S., Jaroniec, M. & Qiao, S. Z. Graphitic Carbon Nitride Nanosheet–Carbon Nanotube Three-Dimensional Porous Composites as High-Performance Oxygen Evolution Electrocatalysts. *Angew. Chem. Int. Ed.* **53**, 7281-7285, (2014).

22 Zhuang, Z., Sheng, W. & Yan, Y. Synthesis of Monodispere Au@Co_3O_4 Core-Shell Nanocrystals and Their Enhanced Catalytic Activity for Oxygen Evolution Reaction. *Adv. Mater.* **26**, 3950-3955, (2014).

23 Ren, J., Antonietti, M. & Fellinger, T. P. Efficient Water Splitting Using a Simple Ni/N/C Paper Electrocatalyst. *Adv. Energy Mater.* **5**, (2015).

24 Gao, M.-R. *et al.* Nitrogen-Doped Graphene Supported $CoSe_2$ Nanobelt Composite Catalyst for Efficient Water Oxidation. *ACS Nano* **8**, 3970-3978, (2014).

25 Zhao, Y., Nakamura, R., Kamiya, K., Nakanishi, S. & Hashimoto, K. Nitrogen-doped carbon nanomaterials as non-metal electrocatalysts for water oxidation. *Nat. Commun.* **4**, 2390, (2013).

26 Gao, M. *et al.* Efficient water oxidation using nanostructured α-nickel-hydroxide as an electrocatalyst. *J. Am. Chem. Soc.* **136**, 7077-7084, (2014).

27 Li, Z., Dou, X., Zhao, Y. & Wu, C. Enhanced oxygen evolution reaction of metallic nickel phosphide nanosheets by surface modification. *Inorg. Chem. Front.* **3**, 1021-1027, (2016).
